# Characterization of the Static, Creep, and Fatigue Tensile Behavior of Basalt Fiber/Polypropylene Composite Rods for Passive Concrete Reinforcement

**DOI:** 10.3390/polym13183136

**Published:** 2021-09-16

**Authors:** Jonathon Tanks, Kimiyoshi Naito, Hisai Ueda

**Affiliations:** 1 Research Center for Structural Materials, National Institute for Materials Science, 1-2-1 Sengen, Ibaraki, Tsukuba 305-0047, Japan; naito.kimiyoshi@nims.go.jp; 2 Department of Aerospace Engineering, Tohoku University, 6-6-1 Aramaki-aza-Aoba, Miyagi, Sendai 305-0047, Japan; 3 Innovative Composite Materials Research and Development Center, Kanazawa Institute of Technology, 2-2 Yatsukaho, Ishikawa, Hakusan 924-0838, Japan; h-ueda@neptune.kanazawa-it.ac.jp

**Keywords:** thermoplastic composite, basalt fiber, fatigue properties, creep properties

## Abstract

Fiber-reinforced polymer (FRP) composites are becoming more frequently adopted as so-called “corrosion-resistant” concrete reinforcement materials due to their excellent mechanical properties and formability. However, their long-term reliability must be thoroughly investigated in order to understand failure mechanisms and to develop service life models. This study is on the mechanical properties of a prototype basalt fiber-reinforced polypropylene (BFPP) rod under quasi-static and sustained loading. Static strength and modulus at elevated temperatures do not decrease significantly, but the variability in strength increases with temperature, as shown by a Weibull analysis. Creep behavior is typical of unidirectional FRP, where the creep rupture strength follows a power law. Fatigue at various stress ratios *R* reveals the sensitivity of composite strength to the matrix damage, which increases at lower values of *R* (i.e., higher stress amplitudes). These results are discussed in the context of service life and concrete structure design guidelines.

## 1. Introduction

Reinforced and prestressed concrete is the most common structural system in the world, given its low cost per unit weight and formability [1]. However, corrosion of the steel reinforcing/prestressing materials such as bars and strands leads to concrete cracking due to internal pressure caused by low-density iron oxide byproducts; costly repairs and even replacement can outweigh the initial construction costs [1,2,3]. Epoxy-coating carbon steel and stainless steel reinforcing products are obvious alternatives, but fiber-reinforced polymer (FRP) composites are becoming more frequently adopted as so-called “corrosion-resistant” concrete reinforcement materials due to their excellent mechanical properties, low density, and resistance to galvanic corrosion [4,5,6,7,8]. Numerous studies on FRP for structural reinforcement are reported every year, covering topics such as environmental durability [9,10,11,12,13,14,15,16] and material mechanics [16,17].

Considering the time scale of service life for a concrete structure, long-term durability and reliability of the reinforcing materials are extremely important. Thus, creep and fatigue studies provide crucial data and analysis regarding the long-term performance of FRP reinforcement and methods for service life prediction. Extensive work has been conducted on three common types of composites: carbon (CFRP), glass (GFRP), and aramid (AFRP) [18,19,20,21,22,23,24,25,26,27]. Extrapolating available experimental data to at least 50-year service periods, numerous studies found that, while CFRP exhibits excellent creep and fatigue resistance (less than 20% decrease in strength), GFRP and AFRP tend to show drastic deterioration of mechanical properties (as much as 90% decrease in strength) [18,19,21,22,23,24,25,26,27]. While CFRP is clearly superior in terms of mechanical reliability and durability, its high cost is a hindrance toward its widespread use in infrastructure compared with cheaper materials such as GFRP [4,8,22].

Basalt fibers, which are drawn from basalt rocks and come at relatively low cost, have recently gained more attention as an alternative to glass fibers due to having superior mechanical properties [28,29,30,31]. The creep behavior of basalt FRP (BFRP)—especially containing epoxy matrices—has been investigated on cylindrical bars [32,33], and fatigue has been studied in various geometries with respect to failure mechanisms [34,35,36,37], stress ratios [38], matrix type [39], and environmental conditions [40]. Several studies found significant reductions in stiffness over the fatigue life caused by increased matrix damage and fiber rupture at longer cycles, with a 10^7^-cycle fatigue strength around 70–75% of the initial static strength [37,38,39]. However, the effect of stress ratio *R* (i.e., the ratio of minimum and maximum applied stresses in a sinusoidal cycle) has not been investigated in great depth, as only *R* = 0.1, 0.5, and 0.8 were reported [37,38]. These studies found that the failure mode of basalt/epoxy laminates changes from interfacial debonding to fiber rupture as *R* decreases. However, as matrix damage and interfacial debonding are largely matrix-dominant, a comparison with other matrix resins is needed.

Furthermore, polypropylene—a low-cost commodity polymer with excellent moisture resistance—has been used as a matrix for GFRP and BFRP in some studies, showing lower strength and modulus than a thermosetting epoxy [15,27,41,42,43,44,45]. The fatigue behavior of glass fiber-reinforced polypropylene (GFPP) was discovered to improve when polypropylene was modified with maleic-anhydride (MA) due to the improved interfacial bonding with the glass fibers, which resulted in a fiber-rupture failure mode rather than interfacial debonding (at *R* = 0.1) [41,42]. In particular, the stiffness degradation was minimal with MA-modified GFPP in stark contrast to the epoxy- or polyester-based GFRP [42,43]. Furthermore, MA-modified PP sizing for BFPP composites showed roughly 20% higher flexural strength compared with neat PP [41,45].

Although glass and basalt share some similarities in their chemical composition, similar studies on the long-term mechanical behavior of basalt fiber-reinforced polypropylene (BFPP) were not found in the literature (to the authors’ knowledge). This paper reports the static, creep, and fatigue properties of a prototype BFPP rod that is intended for passive reinforcement (i.e., non-prestressed) in the concrete foundation of high-speed railway systems. The thermal and mechanical properties of the rapidly produced thermoplastic composite were primarily evaluated by mechanical testing (static and fatigue) and electron microscopy, supported by an analysis of the material service life considering that the influence of stress ratio *R* is presented.

## 2. Materials and Methods

### 2.1. Materials and Preparation

**Figure 1:**
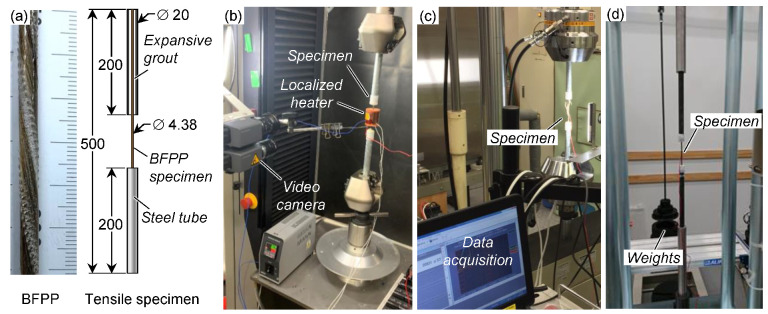
(**a**) Basalt fiber-reinforced polypropylene (BFPP) composite rod (center rod from a seven-wire strand) and tensile specimen diagram (all dimensions in mm), (**b**) static tensile test setup including localized heater, (**c**) fatigue tensile test setup, and (**d**) creep tensile test setup.

The material in this study was a heat-resistant basalt fiber (Nakagawa Sangyo Co., Ltd., Inuyama, Japan) with a matrix made from blended polypropylene (Prima Polymer) and maleic acid-grafted polypropylene (Mitsui Chemical, Tokyo, Japan, MA: 0.25 wt%). Straight rods were manufactured by a pultrusion technique at a rate of 15 m/min by the following process: m-PP pellets were melted and transferred to a resin bath via a screw extruder, where the BF rovings were impregnated before being fed through a die and subsequent water cooling, and finally collected in bundles of seven rods, which were twisted to form a stranded cable. The fiber volume fraction was measured as 0.44 (by cross-sectional analysis). The low fiber content is due to the prioritization of excess polypropylene for additional chemical resistance. The cables were slightly twisted (angle of approximately 11°, measured by digital microscope) to retain the diameter and fiber consolidation during pultrusion. The diameter of the straight rods was 4.38 mm, and they were cut to 500 mm lengths for tensile testing. All tensile test specimens (static, creep, and fatigue) were prepared by fixing steel tubes (inner diameter 16 mm and length 200 mm) to both ends by an expansive grout (Bristar 100, non-explosive demolition grout) and left to cure for at least three days before testing (Figure 1a); alignment was ensured by enclosing the tubes with PVC caps having concentric holes (diameter ~4.5 mm), and the specimens were secured in steel angles. It was found that the expansive pressure from the grout could cause early fatigue failure inside the tubes, so a polyurethane coating was applied in the gripping region to more evenly distribute the gripping pressure [46].

### 2.2. Characterization Methods

Static tensile tests were conducted at a displacement rate of 1 mm/min on a universal testing machine (Autograph AGX, Shimadzu, Kyoto, Japan; Figure 1b), and strain was measured by foil gauges and a video displacement system (TRViewX, Shimadzu). In addition to room temperature (~23 °C), higher temperatures of 80 (±2) and 120 (±2) °C were applied through a local heating device (with internal K thermocouple) to avoid slippage in the gripping area. Once the testing temperature was reached, the specimen was allowed to equilibrate for one hour before conducting the tensile test. Ten specimens were tested for all temperatures.

Fatigue tensile tests were conducted at room temperature on a servohydraulic testing machine (Servopulser, Shimadzu; Figure 1c) under force control at a frequency of 10 Hz and stress ratios *R* (=*σ_min_*/*σ_max_*) of 0.1, 0.3, 0.5, 0.7, and 0.9; this frequency was selected because it allows for faster turnover of the testing equipment without introducing internal heating effects [39,47], and no particular frequency is specified by ASTM [48]. Strain was measured by foil gauges connected to a datalogger. One specimen was tested at each stress level, with run-out (endurance limit) set to *N_e_* = 10^7^ cycles. Creep tests (*R* = 1) were also conducted at room temperature on a lever-arm creep machine (Shimadzu; Figure 1d) at five different stress levels; 2000 h was chosen as the termination time.

To further investigate the thermomechanical properties of the BFPP, dynamic mechanical analysis (DMA 7100, Hitachi Hi-Tech, Tokyo, Japan) was conducted in flexural mode and differential scanning calorimetry (DSC 7020, Hitachi Hi-Tech) was performed using roughly 7–10 mg of neat polymer blend (called m-PP) over a range of 30–200 °C at a heating rate of 10 °C/min (heat–cool–heat). Fracture surfaces were observed by scanning electron microscope (Quanta 200, FEI, Hillsboro, OR, USA).

## 3. Results and Discussion

### 3.1. Thermomechanical Properties

**Figure 2:**
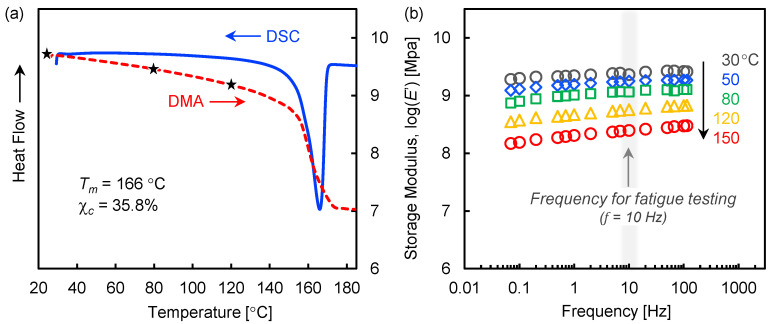
(**a**) Thermal properties of a m-PP matrix and BFPP measured by DSC and DMA, and (**b**) frequency dependence of the storage modulus of BFPP measured by DMA.

Thermal analysis of the neat polymer by DSC (Figure 2a) revealed the melting temperature *T_m_* to be 166.0 °C (onset around 145.7 °C) and a crystallinity χ_c_ of 35.8%, which are typical values for m-PP [43]. The storage modulus *E*’ of the BFPP as measured by DMA (*f* = 10 Hz) showed typical behavior, with a gradual reduction in stiffness followed by a sharp drop at the onset of melting; no influence from the fibers on PP melting was detected. Reductions in *E*’ of 35.1% and 66.2% were observed at the selected static tensile test temperatures of 80 and 120 °C, respectively (marked by star symbols). A linear dependence of log(*E*’) vs. log(*f*) can be seen in Figure 2b, consistent with the literature [49].

### 3.2. Static Properties at Elevated Temperatures

**Figure 3:**
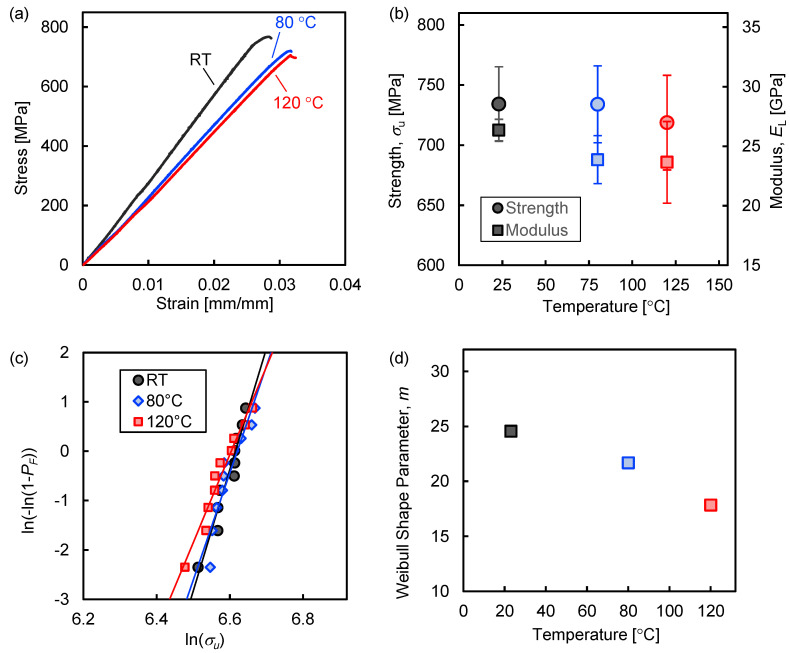
(**a**) Representative stress–strain curves for tensile tests, (**b**) a summary of the tensile properties at each temperature; (**c**) Weibull plot of tensile strengths, and (**d**) Weibull shape parameters at each temperature.

The static tensile strength and modulus at room temperature were 733.5 MPa and 26.7 GPa, respectively; the constitutive behavior was mostly linearly elastic with a small inelastic portion near failure (Figure 3a). At higher temperatures, the strength shows an insignificant decrease (<2%) even at 120 °C, while the modulus decreases slightly (9.5% and 10% at 80 and 120 °C, respectively) but not with statistical significance. Tensile properties at each temperature are listed in Table 1 and shown in Figure 3b. Despite significant softening occurring in the m-PP matrix at higher temperatures, the fiber-dominant properties of the unidirectional composite do not significantly decline. This means that, although the tensile strength of the BFPP is not comparable with other standard materials such as GFRP or CFRP, the performance retention at sub-melting temperatures shows promise for thermoplastic composite reinforcing rods.

The tensile strengths at each temperature were fit to a two-parameter Weibull distribution (total of *n* specimens, with *i* from 1 to *n*) [50]:

(1) $$\ln\left[\ln\left(\frac{1}{1-P_{F}}\right)\right]=m\left[\ln\left(\sigma_{u}\right)-\ln\left(\sigma_{0}\right)\right]$$

(2) $$P_{F}=\frac{i}{n+1}$$

where $P_{F}$ is the cumulative probability of failure at the applied tensile stress $\sigma_{u}$, *m* is the Weibull shape parameter, and $\sigma_{0}$ is the characteristic stress or Weibull scale parameter. The higher the value of *m*, the lower the probability of fracture at stresses approaching the mean. Figure 3c shows the Weibull distributions for each temperature, which revealed that *m* decreases linearly with increasing temperature (Figure 3d). The polymer matrix softens at higher temperatures, which ultimately reduces the interfacial properties and increases the scatter in strength values. This has implications for BFPP rods used at elevated temperatures, such as the curing of prestressed concrete, which takes place at ~60 °C for 6–12 h [51]. While concrete curing temperatures and service temperatures are not likely to exceed 80 °C in most cases and, therefore, the mean tensile strength is not expected to decrease significantly, the decrease in *m* suggests that failure can occur more frequently at stresses well below the statistical mean and should be accounted for when considering safety factors in design and construction.

**Figure 4:**
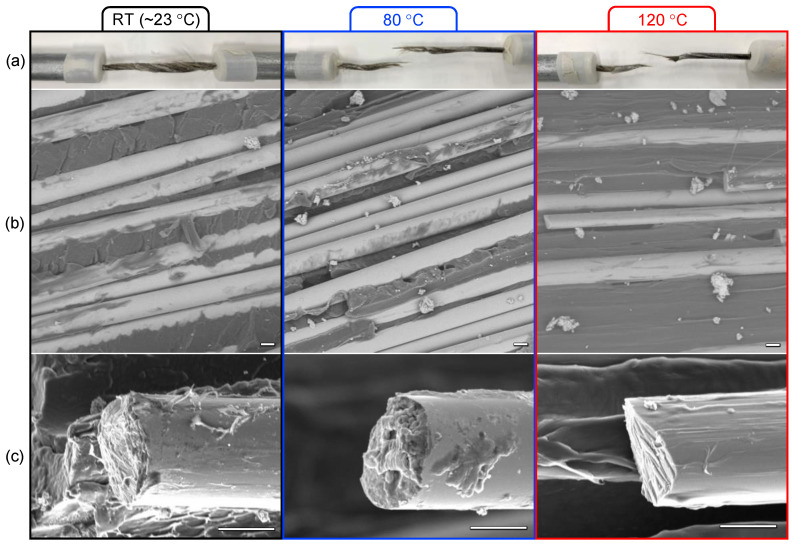
Observation of fractured specimens at higher temperatures: (**a**) specimen appearance after tensile testing, (**b**) fiber and matrix condition, and (**c**) fracture surface of individual fibers (all scale bars are 10 μm).

Specimens at room temperature exhibited a typical broom-like failure mode, where the fiber twist is clearly visible (Figure 4a). At room temperature, failure mainly consists of cohesive failure, indicated by matrix hackles and a residual matrix adhered to the exposed fiber surfaces. Conversely, the failure mode became more localized at higher temperatures due to the softening of the polypropylene, which reduces brittle fracture and increases the probability of localized fiber stress concentrations during loading. Micrographs of the fracture surfaces (Figure 4b) show more fiber fragmentation and debonding as temperature increases, which is an expected outcome considering the significant softening of the matrix discussed in Section 3.1. This corroborates the results of the Weibull analysis regarding the increase in scatter despite small changes in the mean. Fracture surfaces of individual fibers (Figure 4c) become slightly more angular at higher temperatures, but no significant difference was noted; this is expected for fibers marketed as heat-resistant.

### 3.3. Creep Behavior (R = 1) at Room Temperature

**Figure 5:**
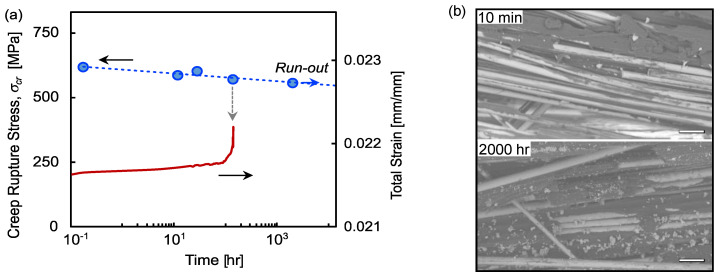
(**a**) Creep rupture strength diagram and creep strain until failure, and (**b**) comparison of fracture surfaces for short and long creep lives (scale bars are 50 μm).

Creep can be considered a special case of fatigue where *R* = 1, since there is no fluctuation in the applied load but failure still occurs at stresses below the static tensile strength. The creep rupture stress *σ_cr_* over time is shown in Figure 5a, along with the total strain (elastic + creep) for the stress level *σ_cr_*/*σ_u_* = 0.77. The creep rupture data follows a typical power law:

(3) $$\sigma_{cr}=a{\left(t_{f}\right)}^{b}$$

where *a* and *b* are empirical parameters (listed in Table 2), and *t_f_* is the time-to-failure. The creep endurance stress level (i.e., terminated at 2000 h) was *σ_e,cr_*/*σ_u_* = 0.75, but extrapolating with Equation (3) to 10^6^ h (114 years) yields *σ_e,cr_*/*σ_u_* = 0.71. The strain also shows typical creep behavior, with a steady state region in the short-term and a sudden increase shortly before failure. Strain data from other specimens were not recoverable, but this specimen shows the anticipated behavior for FRP.

The micrographs in Figure 5b show more damage in the resin for longer creep durations, comparing 10 min with 2000 h. The residual static tensile strength was measured immediately following the termination of the 2000 h creep test, showing no statistically significant change (<0.7%). Creep rupture at short durations despite less matrix damage suggests that internal defects and strength distribution affect the probability of fiber-dominated failure at higher stress levels, since more matrix damage appears to be tolerable at longer durations. Further experimental work is needed to reveal the damage accumulation mechanism under creep loading in BFPP.

### 3.4. Fatigue Behavior (0 < R < 1) at Room Temperature

**Figure 6:**
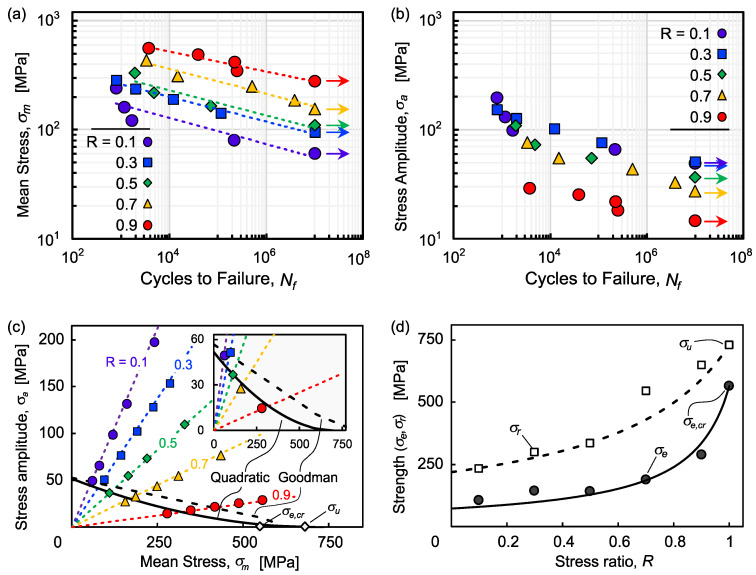
Fatigue life plotted by (**a**) mean stress *σ_m_* and (**b**) stress amplitude *σ_a_*, (**c**) fatigue life diagram comparing Goodman and quadratic (convex) failure criterion (Equation (6)), and (**d**) endurance limit *σ_e_* and residual strength *σ_r_* corresponding to different stress ratios *R*.

Figure 6a shows the S–N curves in terms of mean stress *σ_m_* for each value of *R*, while Figure 6b shows the S–N curves in terms of stress amplitude *σ_a_*. Replicates were not tested for each stress level so a statistical analysis could not be performed, but the data appear to follow a power law similar to Equation (3):

(4) $$\sigma_{m}=a{\left(N_{f}\right)}^{b}$$

where *a* and *b* are empirical parameters, and *N_f_* is the cycles to failure; *a* and *b* are listed in Table 2, along with the maximum stress at the endurance limit (*σ_e,max_*/*σ_u_*). It is clear that a smaller stress amplitude (higher *R*) results in a higher tolerable mean stress for a given fatigue life while a higher mean stress results in a lower tolerable stress amplitude for a given fatigue life. It is easy to understand that a smaller stress amplitude creates less damage and thus correlates to higher mean stress. From a reliability perspective, for a given mean stress, a higher stress amplitude translates to a higher probability that the maximum stress approaches the mean static strength, as shown by the Weibull distribution (Section 3.2).

This is especially clear in the fatigue failure diagram in Figure 6c, where the relationship between stress amplitude and mean stress are linear for each value of *R*, and the fatigue endurance (run-out) line is formed by the smallest values from each series. A variety of fatigue failure criteria has been developed for metal alloys and applied to composite materials [52,53,54], most notably the Goodman criterion, which is given by the following:

(5) $$\sigma_{a}=\sigma_{w}\left(1-\frac{\sigma_{m}}{\sigma_{u}}\right)$$

where *σ_w_* is an upper bound on the stress amplitude when the mean stress approaches zero—i.e., fully reversed loading. However, this criterion clearly does not fit the experimental data for BFPP, as the fatigue limit is overestimated. Other common criteria such as Soderberg and Gerber are also more suitable for metal alloys, which exhibit a yielding behavior, and thus, the fatigue limit is affected by plastic deformation [52]. A new empirical criterion is introduced here to more accurately represent the experimental results of this study:

(6) $$\sigma_{a}=\sigma_{w}+A{\left(\sigma_{m}\right)}^{2}+B\sigma_{m}$$

where the coefficients *σ_w_*, *A*, and *B* are defined by the following:

(7) $$\sigma_{w}=\frac{2.5{\left(\sigma_{u}\right)}^{2}}{E_{L}}$$

(8) $$A=\frac{2.5}{E_{L}}=\frac{\sigma_{w}}{{\left(\sigma_{u}\right)}^{2}}$$

(9) $$B=-\frac{5\sigma_{u}}{E_{L}}=-2A\sigma_{u}$$

where *E_L_* is the longitudinal tensile modulus. This formulation is expressed in terms of maximum strain energy density at failure for an elastic material. In this context, the fatigue endurance limit *σ_e_* follows a convex surface where a higher mean stress corresponds to an increasingly lower tolerable stress amplitude. The comparison between the Goodman criterion and the proposed quadratic criterion is shown in Figure 6c (enlarged in the inset).

### 3.5. Implications for Service Life and Design

**Figure 7:**
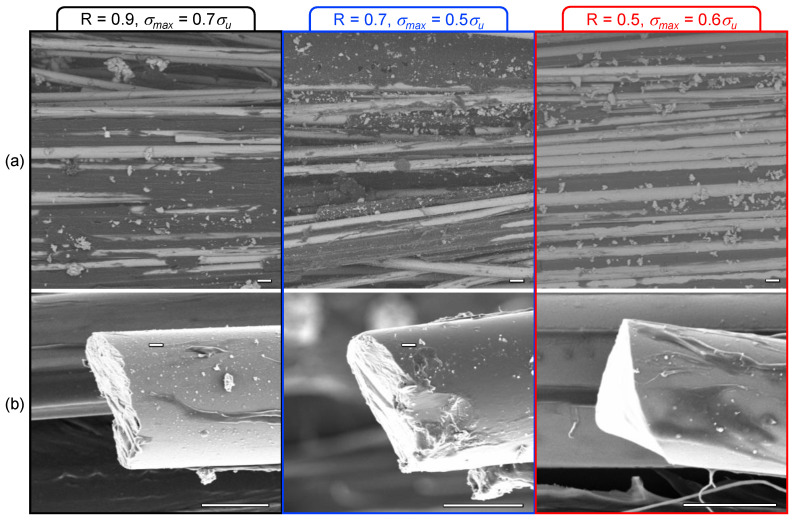
(**a**) Micrographs of fatigue run-out specimens showing more resin damage at lower *R* values and (**b**) fracture surfaces of individual fibers (scale bars are 10 μm).

The endurance limit for each stress ratio is shown in Figure 6d, where *σ_e_* increases with *R* according to the following:

(10) $$\sigma_{e}=1.5\sigma_{w}{\left(\left(1-R\right)+1.5\sigma_{w}\left(\frac{1+R}{2\sigma_{cr,e}}\right)\right)}^{-1}$$

where *σ_e,cr_* is the creep endurance strength (i.e., *σ_e_* at *R* = 1). The residual static strength *σ_r_* was measured from specimens after reaching run-out (*N* = 10^7^ cycles) and was found to increase with *R* according to the following:

(11) $$\sigma_{r}=5\sigma_{w}{\left(\left(1-R\right)+5\sigma_{w}\left(\frac{1+R}{2\sigma_{u}}\right)\right)}^{-1}$$

which is similar to Equation (10) except that the static tensile strength is used as the upper bound instead of the creep endurance strength. As mentioned above, more damage is accumulated at higher stress amplitudes (lower *R*), so the residual static strength is significantly reduced. Conversely, the post-creep (*R* = 1) residual static strength is unchanged. This is supported by the micrographs in Figure 7, which show significant resin damage for lower values of *R* while higher values do not differ from static tensile fracture surfaces. This deviates from results for basalt/epoxy composites [38,39], which is assumed to be caused by the difference in stiffness and ductility between epoxy and polypropylene. However, these results may extend the observations of the effect of limited ductility for toughened vinylester-based BFRP [39]. No significant reduction in stiffness (*E*/*E*_0_) was observed for any of the loading conditions in this study due to the fiber-dominant behavior of unidirectional composites. Further experimental and analytical investigation is needed to fully characterize and quantify damage under sustained loading.

Existing standards and design guides for FRP concrete reinforcement do not include BFRP and nearly all referenced data come from brittle thermoset matrix composites, so we reference the guidelines made for GFRP as it is the most similar to BFRP. The American Concrete Institute (ACI) published the ACI 440.1R-15 “Guide for the Design and Construction of Structure Concrete Reinforced with Fiber-Reinforced Polymer (FRP) Bars”, which is currently the most comprehensive document on the topic [55]. Section 7.4 of ACI 440.1R-15 addresses creep rupture and fatigue limits, where a maximum long-term stress of *σ_max_* = 0.2*σ_u_* is recommended for GFRP. Referring to the endurance limits for each *R* in Table 2, BFPP exhibits similar values ranging from 0.15*σ_u_* to 0.40*σ_u_* (for *R* = 0.1 and 0.9, respectively) and 0.71 for creep (*R* = 1). Although ACI 440.1R-15 does not mention stress ratios, in-service structures experience variable loading scenarios that make life prediction complex, which is why conservative stress limits are needed for safe designs. More experimental data and theoretical analysis are needed to better understand the fatigue and creep behavior of basalt fiber/thermoplastic composites and to develop accurate service life prediction models.

## 4. Conclusions

This paper reports the static and fatigue tensile behavior of a novel basalt fiber/polypropylene composite rod for concrete reinforcement. In particular, the elucidation of the effect of stress ratio on fatigue life of BFPP, and the proposal of a failure criterion and the relationship between endurance limit and residual strength are major contributions of this study. To summarize, the static tensile properties at elevated temperatures (*T* < *T_m_*) decreased slightly but the fiber-dominant nature of unidirectional composites resulted in a smaller decrease than expected based on the neat resin’s thermal properties. Rather, the most notable change was an increase in the variability in strength as temperature increased, as indicated by a decrease in the Weibull shape parameter. Fatigue behavior was similar to other FRP (particularly GFRP) in terms of the general relationship between stress level and fatigue life; however, the endurance limit deviated from standard failure criteria such as the Goodman equation, instead being better described by a strain energy density-based quadratic (convex) function. Additionally, we found that a higher stress ratio *R* (i.e., lower stress amplitude) resulted in a higher residual static strength after run-out, with no change for creep loading (2000 h run-out). A lower stress amplitude corresponds to less damage accumulation in the m-PP matrix, although the damage mechanism for creep (*R* = 1) is yet unclear. Several equations were introduced to describe the fatigue endurance limit and residual strength, showing good agreement with experimental data. The strength and stiffness of this prototype cable are insufficient for prestressed concrete applications, but it is a promising candidate material for passive concrete reinforcement due to its durability and low cost. Although further investigation is needed to thoroughly characterize fatigue damage mechanisms and to accurately predict fatigue life, these results suggest that the BFPP material in this study is comparable with other FRP and seems conforms to ACI 440.R-15.

## Figures and Tables

**Table 1:** Static tensile properties of BFPP rods.

Temperature (°C)	*σ_u_* (MPa)	*E_L_* (GPa)	*m* (-)
~23	733.5	26.7	24.55
80	733.5	23.9	21.67
120	718.8	23.7	17.83

**Table 2:** Fatigue life parameters of BFPP rods.

*R*	*a*	*b*	*σ_e,max_*/*σ_u_*
0.1	1211.1	−0.092	0.15
0.3	1054.0	−0.116	0.20
0.5	663.0	−0.115	0.20
0.7	575.6	−0.114	0.25
0.9	391.5	−0.121	0.40
1.0	607.8	−0.011	0.75 *

* Extrapolation to 10^6^ h gives 0.71.

## Data Availability

Requests for experimental data may be considered on a case-by-case basis.
